# Prediction Model for Cereblon Expression in Bone Marrow Plasma Cells Based on Blood Markers in Multiple Myeloma Patients

**DOI:** 10.3389/fonc.2021.687361

**Published:** 2021-07-14

**Authors:** Byung-Hyun Lee, Ka-Won Kang, Min Ji Jeon, Eun Sang Yu, Dae Sik Kim, Se Ryeon Lee, Hwa Jung Sung, Yong Park, Chul Won Choi, Byung Soo Kim

**Affiliations:** ^1^ Department of Internal Medicine, Korea University College of Medicine, Anam Hospital, Seoul, South Korea; ^2^ Department of Internal Medicine, Korea University College of Medicine, Guro Hospital, Seoul, South Korea; ^3^ Department of Internal Medicine, Korea University College of Medicine, Ansan Hospital, Gyeonggi-do, South Korea

**Keywords:** cereblon, immunomodulatory therapy, multiple myeloma, nomograms, prognosis

## Abstract

**Background:**

Cereblon (CRBN) is a direct target of immunomodulatory drugs (IMiDs) and is known to be sensitive and responsive to IMiD therapy. We evaluated CRBN expression in bone marrow plasma cells and analyzed whether CRBN expression was associated with multiple myeloma prognosis. Lastly, we developed a nomogram model for predicting high CRBN expression based on clinically significant blood markers.

**Methods:**

We evaluated 143 multiple myeloma patients (internal dataset) who underwent bone marrow examinations. For evaluating the prognostic ability of the nomogram model, two external cohorts (235 patients in external dataset 1 and 156 patients in external dataset 2) were analyzed. The expression of CRBN in bone marrow aspirate samples was evaluated using immunohistochemistry. High CRBN expression was defined as the study-defined H-score ≥6.

**Results:**

In the high CRBN group, the median progression-free survival (PFS) and overall survival (OS) of patients receiving the IMiD-based therapy and non-IMiD therapy were 29 and 10 months for PFS, and NR (not reached) and 54 months for OS, respectively. IMiD-based therapy was significantly associated with better PFS and OS outcomes. High CRBN expression was independently predicted by female sex, high serum free-light chain (FLC) ratio, higher serum M-protein level, and higher β2-microglobulin level. Based on these results, we constructed a new nomogram model to predict high CRBN expression and the effectiveness of IMiD therapy in multiple myeloma.

**Conclusion:**

This nomogram could improve the prognostic evaluation of myeloma patients exhibiting high CRBN expression treated with IMiD therapy and might help provide personalized treatment strategies to clinicians.

## Introduction

Cereblon (CRBN) is a multifunctional protein located in the cytoplasm, nucleus, and peripheral membranes of the human tissues (1). CRBN is closely related to the proliferation and metabolism of normal cells as well as tumor cells and is responsible for antiproliferative activities of immunomodulatory drugs (IMiDs) (2). CRBN expression affects cellular metabolism and can lead to several diseases including cardiovascular disease, fatty liver, and neurodegenerative diseases (e.g. Alzheimer’s disease) in the absence of IMiD treatment (1, 3). CRBN is a direct target of IMiDs and is associated with patient sensitivity and response to IMiD therapy; thus, providing a theoretical basis for individual clinical treatments (1). IMiDs have a variety of mechanisms of action including angiogenesis inhibition, anti-inflammatory properties, reduction of cell-to-cell interactions, and osteoclast growth reduction (4). IMiDs (thalidomide, lenalidomide, or pomalidomide) provide the backbone of multiple myeloma treatment for both newly diagnosed and relapsed diseases (5–7) including maintenance therapy (8) and can be combined with new treatment strategies that themselves have immunomodulatory activities (9). Previous studies reported that CRBN is required for the anti-myeloma effect of thalidomide and its derivatives, lenalidomide and pomalidomide, which are referred to as CRBN-binding small molecules (10). The significance of CRBN in modulating the anti-myeloma effects of IMiDs was reported by a study in which knockdown of CRBN decreased viability of myeloma cells and induced resistance to IMiD therapy (11).

The clinical and prognostic significance of CRBN in multiple myeloma patients have been previously reported in several studies. Some showed that the higher CRBN gene expression is associated with improved clinical responses in patients with multiple myeloma receiving IMiD-based treatments (12–14). Furthermore, low CRBN expression has been shown to significantly decrease the progression-free survival (PFS) and overall survival (OS) of patients receiving IMiD-based therapy (14). Another study reported that higher expression of the CRBN gene is associated with better survival in multiple myeloma patients treated with thalidomide maintenance therapy (15). In a study evaluating CRBN protein expression using immunohistochemistry (IHC), the CRBN expression was associated with better treatment response in IMiD-based treatment (16). Thus, CRBN expression appears to have prognostic significance for patients treated with IMiDs, and CRBN expression could be used as a predictor of treatment responses.

This study aimed to evaluate expression of CRBN in bone marrow plasma cells and investigate whether CRBN is related to the prognosis of multiple myeloma. Then, based on the study results, we constructed a nomogram model for predicting high CRBN expression using blood markers. We think that this nomogram could identify multiple myeloma patients who can benefit from IMiD-based therapy.

## Methods

### Patients

We retrospectively evaluated patients with newly diagnosed multiple myeloma who underwent bone marrow examination at the Korea University Anam Hospital. The cohort included 170 multiple myeloma patients diagnosed between January 2010 and May 2019. Among them, 27 patients were excluded because their bone marrow specimens were unavailable; thus, 143 bone marrow specimens obtained at the time of diagnosis were examined (internal dataset). In addition, for evaluating the prognostic ability of the nomogram model, medical records of the internal and two external cohorts were reviewed. The first external cohort (external dataset 1) included 259 patients diagnosed between January 2000 and December 2019 at the Korea University Guro Hospital. Among them, 24 patients were excluded because critical medical data were missing; thus, 235 patients were analyzed. The second external cohort (external dataset 2) included 165 patients diagnosed between January 2000 and December 2019 at the Korea University Ansan Hospital. Among them, 9 patients were also excluded because of missing data; thus, 156 patients were analyzed. In survival analyses comparing IMiD and non-IMiD groups, we excluded unsuitable patients who received only supportive care to estimate efficacy of specific treatment against multiple myeloma. Thus, 130 patients (internal dataset), 214 patients (external dataset 1), and 142 patients (external dataset 2) who received any types of drug therapy were included in analyses for survival outcomes.

### IHC

The formalin-fixed and paraffin-embedded bone marrow specimens were sectioned at 4–5 µm thickness and placed on glass slides. Slide sections were baked in an incubator for 1 hour at 60°C to soften the paraffin and deparaffinized with xylene and rehydrated with a graded series of ethanol. Antigen retrieval was performed by boiling in a pressure cooker for 10 min using the sodium EDTA buffer (pH 9.0). Endogenous peroxidase activity was blocked using 3% hydrogen peroxide for 10 min. Primary antibodies for CD138 (Clone EP201, 1:200, Medaysis, Livermore, CA, USA) and CRBN (Clone 2A9D11, 1:200, Proteintech, Rosemont, IL, USA) were used, and the slides were incubated overnight at 4°C. Thereafter, the Polink DS-MR-Hu C1 Kit (GBI Labs, Bothell, WA, USA) was used to double stain for CD138 and CRBN according to manufacturer’s instructions. We calculated the proportion of CRBN expressing CD138^+^ plasma cells with respect to all plasma cells, and estimated proportion score of positive-staining plasma cells (0, none; 1, < 1/100; 2, 1/100 to 1/10; 3, 1/10 to 1/3; 4, 1/3; to 2/3; and 5, >2/3). The intensity score was calculated based on the average intensity of the positive-staining plasma cells (0, none; 1, weak, 2, intermediate; and 3, strong). The proportion and intensity scores were added to obtain a total score (H-score), which ranged from 0 to 8, according to a previously described method (17). We defined the optimal cut-off value for high CRBN expression based on the absolute value of the maximal log rank statistic in patients who received IMiD-based treatments, as proposed by Contal and O’Quigley (18). Human liver tissue was used as a positive control. Each slide was examined by two investigators, including one pathologist and one hematologist, and discrepancies were discussed until a consensus was reached. An optical microscope (DS-Fi2; Nikon Metrology, Tokyo, Japan) was used for image acquisition and examinations.

### ELISA

Bone marrow samples were lysed with radioimmunoprecipitation assay lysis buffer and 20 μg of total protein from each sample was quantified using the Bradford assay. The CRBN protein in each cell lysate was quantitated using human CRBN ELISA kit (MyBioSource, San Diego, CA, USA), according to the manufacturer’s protocol. All samples were tested in duplicate (10 μg of total protein used for each test) and the mean values were used for further analysis.

### Predictive Nomogram

A predictive nomogram was developed using the logistic regression method based on the internal dataset. A bootstrap aggregating method was used to validate the model and correct for potential overfitting. The calibration curves were assessed by plotting the actual probability against the nomogram-predicted probability based on bootstrapping with 1,000 resamples. Receiver operating characteristic (ROC) curves and area under the curve (AUC) analyses with 1,000 bootstrap replicates were used for evaluating the performance and prediction accuracy of the nomogram model. Optimal cut-off point for estimating high CRBN expression using the nomogram was identified as the point at which the value of the cross validated AUC was maximal.

### Statistical Analysis

Categorical variables were evaluated using the chi-squared or Fisher’s exact tests, and continuous variables were evaluated using Student’s *t*-test or the Mann–Whitney U test. Pearson correlation coefficient was used to determine the correlation between CRBN expression and clinical parameters. The probability of high CRBN expression was predicted by logistic regression model. The OS was calculated as the time elapsed from the diagnosis to death from any cause. The PFS was calculated as the time elapsed from the start of treatment to disease progression or death from any cause. Survival curves were estimated using the Kaplan-Meier method and differences in the survival distributions were evaluated using the log-rank test. Cox proportional hazard model was used to analyze the associations between survival time and each prognostic variable. All tests were two-sided, and *P*-values <0.05 were considered significant. The statistical analyses were performed using SPSS statistics version 25.0 software (IBM Corporation, New York, NY, USA), GraphPad Prism version 9.0.1 software (GraphPad Software Inc., San Diego, CA, USA) and the RStudio software version 1.2.1.

## Results

### CRBN Expression and Clinical Parameters

**Figure 1:**
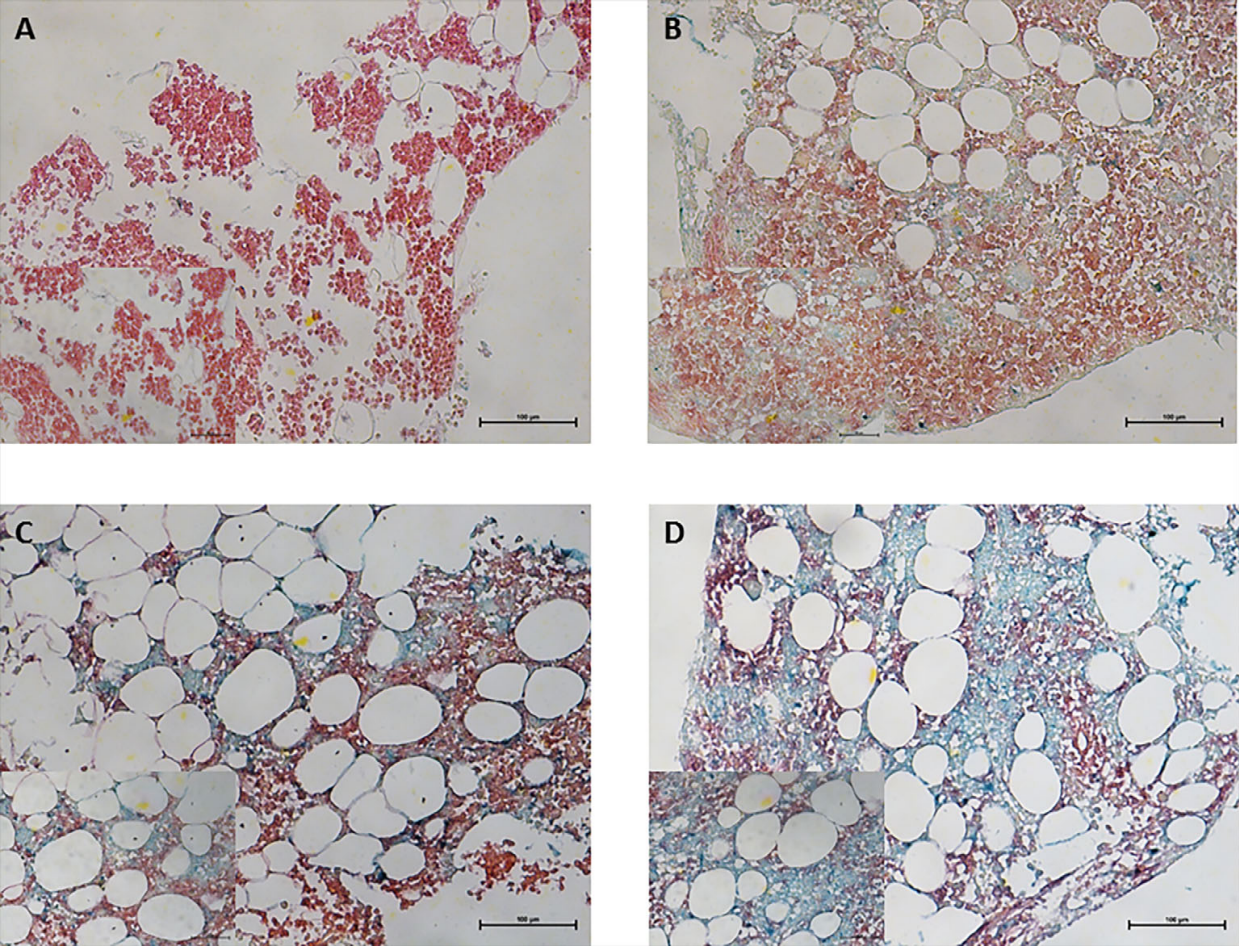
Immunohistochemical staining for CRBN expression in bone marrow plasma cells obtained from multiple myeloma patients. CD138^+^ plasma cells was performed using Permanent Red chromogen (red color) and CRBN-expressing cells were stained using Emerald chromogen (blue-green color). Representative images for the high and low CRBN expression based on H-score. **(A)** 0 and **(B)** 4 for low CRBN expression and **(C)** 6 and **(D)** 8 for high CRBN expression. Top row, original magnification ×200; bottom low inset, original magnification ×400.

We examined the 143 bone marrow specimens for CRBN expression and clinical features. CRBN expression was assessed using a double-staining IHC method (**Figure 1**). The median value of CRBN expression was 25% (interquartile range, 5–75) and mean value was 39.57% [95% confidence interval (CI), 33.89–45.24]. The optimal cut-off point for high CRBN expression was an H-score of 6, according to the statistical method described in section 2.2. Based on the cut-off value of H-score ≥6, we classified 70 patients as having high CRBN expression and 73 patients as having low CRBN expression.

**Figure 2:**
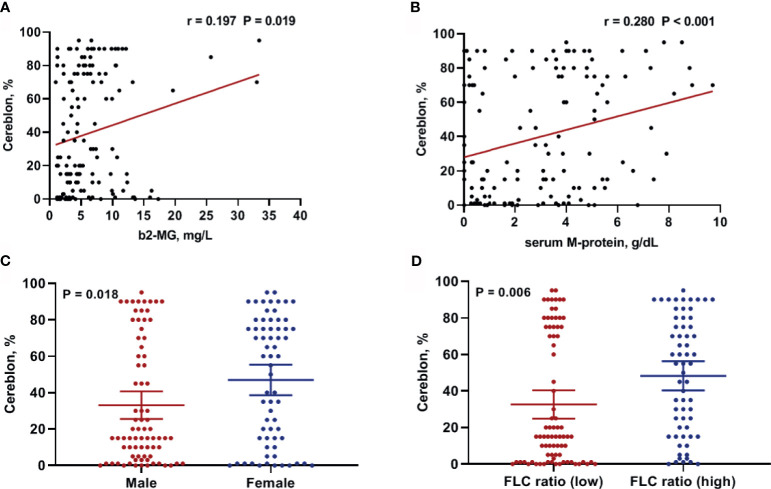
Clinical factors associated with CRBN expression in multiple myeloma patients. Correlation between CRBN expression and **(A)** β2-microglobulin as well as **(B)** serum M-protein levels. Comparison of CRBN expression based on **(C)** sex and **(D)** serum FLC ratio. Female patients and patients with high FLC ratio showed significantly higher CRBN expression level compared to male patients and patients with low FLC ratio. FLC, free-light chain.

Next, we examined the correlation between CRBN expression and clinical factors including age, serum and urine M-protein level, β2-microglobulin level, LDH levels, and percentage of bone marrow plasma cells in the internal dataset. The β2-microglobulin level was significantly correlated with CRBN expression (r = 0.197; 95% CI, 0.033–0.350; *P* = 0.019; **Figure 2A**). Furthermore, a significant correlation was observed between serum M-protein level and CRBN expression (r = 0.280; 95% CI, 0.122–0.425; *P* < 0.001; **Figure 2B**). Nevertheless, the other factors were not correlated with CRBN expression. Furthermore, we compared the CRBN expression levels between dichotomous factors including sex, Eastern Cooperative Oncology Group (ECOG) performance status (<2 *vs*. ≥2), serum FLC ratio [high (≤0.01 or ≥100) *vs*. low], cytogenetic abnormalities (high risk *vs.* others), International Staging System (ISS) stage (I + II *vs*. III), and the Revised International Staging System (R-ISS) stage (I + II *vs*. III). High-risk cytogenetics were defined as t(4;14), t(14;16), del(17/17p), TP53 deletion, or chromosome 1 abnormalities, including gain(1q) and del(1p). Female patients showed significantly higher CRBN levels than male patients (mean, 46.98% *vs*. 33.14%; *P* = 0.016; **Figure 2C**), while patients with high FLC ratio showed significantly higher CRBN levels than patients with low FLC ratio (mean, 48.32% *vs*. 32.61%; *P* = 0.006; **Figure 2D**). No significant differences were observed between the other factors.

**Figure 3:**
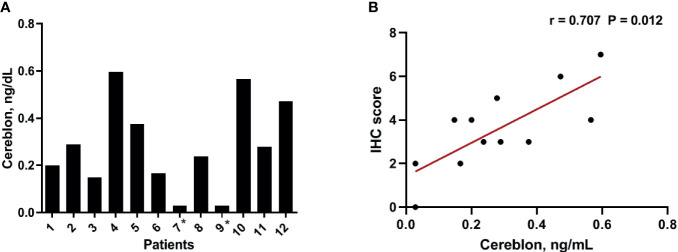
Expression level of CRBN protein. The expression levels were analyzed using ELISA **(A)** The distribution of the CRBN protein levels from 12 patients. **(B)** Correlation between the CRBN expression level measured using ELISA and that obtained based on the H-score. *These values were below a detection limit range and replaced by half of the limit of detection values for statistical analysis.

To validate the results of the CRBN expression based on the H-score, we analyzed CRBN protein levels in the 12 available bone marrow samples using ELISA (**Figure 3A**). Original ELISA data are shown in the **Supplementary Table 1**. We determined the correlation between the CRBN results obtained through the different detection methods, using Spearman correlation analysis. The CRBN expression levels determined from ELISA significantly correlated with that determined based on the H-score from the IHC analysis (r = 0.707; *P* = 0.012; **Figure 3B**).

### Patient Characteristics

The patient characteristics are summarized in **Table 1**. Data from 143 patients, 54 (37.8%) that received IMiD-based therapy, 76 (53.1%) that received non-IMiD therapy, and 13 (9.1%) that received only supportive care were used in this study. IMiD-based therapy was defined as the treatment regimens including thalidomide or lenalidomide and non-IMiD therapy was defined as the treatment regimens that did not include these drugs. Median age was 57 [interquartile range (IQR), 53–62] years in patients who received IMiD-based therapy, 69 (IQR, 66–76) years in patients who received non-IMiD therapy, and 68 (IQR, 63–77) years in patients who received only supportive care. The mean CRBN expression value was 44.65% (95% CI, 35.73–53.57) in the IMiD group, 34.96% (95% CI, 26.90–43.02) in the non-IMiD group, and 45.38% (95% CI, 24.65–66.12) in the supportive care group. Thirty patients (55.6%) from the IMiD group, 33 patients (43.4%) from the non-IMiD group, and 8 patients (61.5%) from the supportive care group exhibited high CRBN expression. Most patients (n = 95; 66.4%) from all groups were in stage II according to the Revised International Staging System classification (n = 38; 70.4% for IMiDs; n = 50; 65.8% for non-IMiDs; and n = 7; 53.8% for supportive care groups). We compared baseline characteristics between the IMiD and non-IMiD groups. Although younger patients were included in the IMiD group (*P* = 0.001), the baseline characteristics were well balanced between the IMiD and non-IMiD groups and no significant differences between the groups were observed among the factors investigated (*P* = 0.110–0.996). Next, we evaluated the prognostic significance of CRBN expression according to treatment strategies.

**Table 1:** Baseline characteristics.

	Total (n = 143)	IMiDs (n = 54)	Non-IMiDs (n = 76)	Supportive (n = 13)	*P*
Age, years	66 (58–72)	57 (53–62)	69 (66–76)	68 (63–77)	0.001
Sex, female	66 (46.2)	28 (51.9)	30 (39.5)	8 (61.5)	0.162
ECOG PS, ≥2	9 (6.3)	3 (5.6)	2 (2.6)	4 (30.8)	0.649
BM plasma cells, %	32.7 (14.9–60.5)	28.9 (12.8–55.3)	37.1 (18.2–65.2)	25.0 (5.1–51.2)	0.193
Serum M-protein, g/dL	2.8 (0.6–4.6)	1.6 (0.3–4.0)	3.1 (0.9–4.8)	4.0 (1.8–6.2)	0.188
>3.0 g/dL	70 (49.0)	21 (38.9)	40 (52.6)	9 (69.2)	0.122
Serum FLC ratio					
≤0.01 or ≥100	63 (44.1)	26 (48.1)	33 (43.4)	4 (30.8)	0.594
ß2-microglobulin, mg/L	4.64 (3.05–8.08)	4.52 (2.80–7.84)	4.97 (3.09–9.10)	4.39 (2.33–7.80)	0.179
≥5.5 mg/L	58 (40.6)	20 (37.0)	34 (44.7)	4 (30.8)	0.380
LDH, IU/L	383 (303–492)	367 (284–482)	397 (309–506)	369 (295–422)	0.755
≥ Upper normal range	56 (39.2)	22 (40.7)	31 (40.8)	3 (23.1)	0.996
Cytogenetic abnormalities					
High risk*	37 (25.9)	14 (25.9)	21 (27.6)	2 (15.4)	0.648
ISS					
Stage I	30 (21.0)	16 (29.6)	11 (14.5)	3 (23.1)	0.110
Stage II	55 (38.5)	18 (33.3)	31 (40.8)	6 (46.2)	
Stage III	58 (40.6)	20 (37.0)	34 (44.7)	4 (30.8)	
R-ISS					
Stage I	17 (11.9)	8 (14.8)	6 (7.9)	3 (23.1)	0.179
Stage II	95 (66.4)	38 (70.4)	50 (65.8)	7 (53.8)	
Stage III	31 (21.7)	8 (14.8)	20 (26.3)	3 (23.1)	
mSMART 3.0					
Standard	88 (61.5)	35 (64.8)	44 (57.9)	9 (69.2)	0.426
High	55 (38.5)	19 (35.2)	32 (42.1)	4 (30.8)	

Data are shown as number (percentage) or median (interquartile range).

Statistical differences were calculated between the IMiD and non-IMiD groups.

BM, bone marrow; ECOG, Eastern Cooperative Oncology Group; FLC, free light chain; IMiD, immunomodulatory drug; ISS, International Staging System; LDH, lactate dehydrogenase; mSMART, Mayo Stratification of Myeloma and Risk-Adapted Therapy; PS, performance status; R-ISS, Revised International Staging System.

*High-risk cytogenetics were defined as t(4;14), t(14;16), del(17/17p), TP53 deletion, or chromosome 1 abnormalities including gain(1q) and del(1p).

### Survival Analyses for Immunotherapy According to CRBN Expression

**Figure 4:**
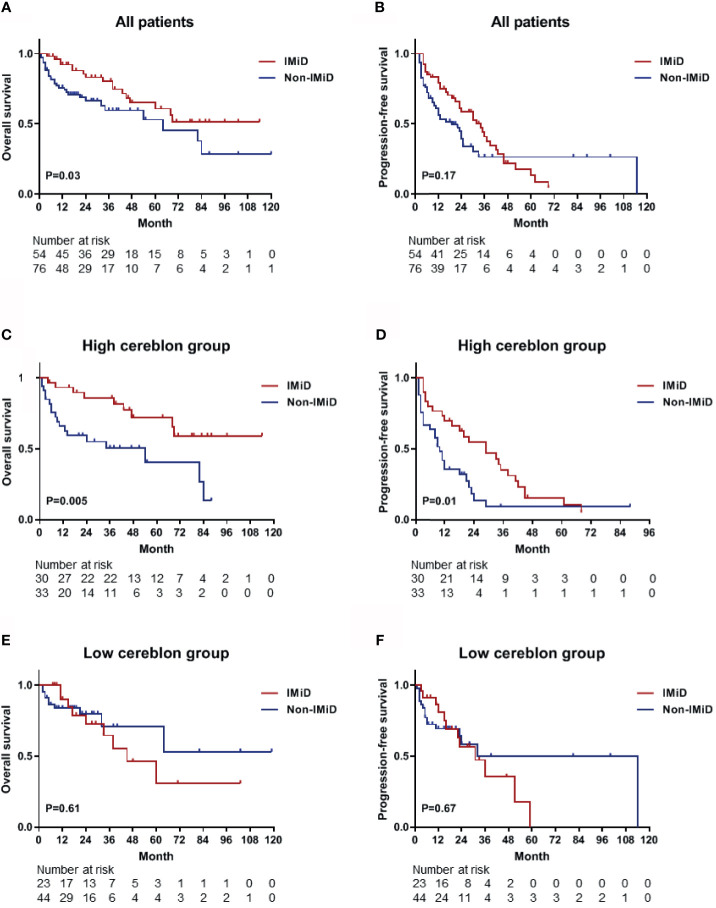
Kaplan–Meier survival curves for OS and PFS. **(A, B)** The OS and PFS curves for all patients (n = 130) who received any type of drug therapy. The median OS and PFS were NR (not reached) and 34 months in the IMiD group and 64 and 19 months in the non-IMiD group, respectively. **(C, D)** The OS and PFS curves for the high CRBN group (n = 63). The median OS (NR *vs*. 54 months) and PFS (29 *vs*. 10 months) were significantly longer in the IMiD group compared to the non-IMiD group. **(E, F)** The OS and PFS curves for the low CRBN group (n = 67). The median OS and PFS of the IMiD and non-IMiD groups were 45 months and NR for OS and 31 and 32 months for PFS, respectively. IMiD, immunomodulatory drug; OS, overall survival; PFS, progression-free survival.

From the 143 patients, 130 patients who received any type of drug therapy were analyzed. The median OS of patients who received IMiD-based therapy and non-IMiD therapy were NR (not reached) and 64 months, respectively. A significant difference was observed for OS between the two groups [hazard ratio (HR), 1.862; 95% CI, 1.044–3.320; *P =* 0.03; **Figure 4A**]. The median PFS of patients who received IMiD-based therapy and non-IMiD therapy were 34 and 19 months, respectively and there was no significant difference between the two groups (HR, 1.343; 95% CI, 0.868–2.076; *P =* 0.17; **Figure 4B**). In patients with high CRBN expression, the median OS and PFS of patients who received IMiD-based therapy and non-IMiD therapy were NR and 54 months for OS and 29 and 10 months for PFS, respectively. IMiD-based therapy was significantly associated with better OS (HR, 2.881; 95% CI, 1.338–6.203; *P* = 0.005; **Figure 4C**) and PFS (HR, 1.911; 95% CI, 1.085–3.366; *P* = 0.01; **Figure 4D**). In patients with low CRBN expression, the median OS and PFS of patients who received IMiD-based and non-IMiD therapy were 45 months and NR, for OS and 31 and 32 months, for PFS, respectively. However, IMiD-based therapy was not significantly associated with better OS (HR, 0.795; 95% CI, 0.319–1.978; *P* = 0.61; **Figure 4E**) and PFS (HR, 0.854; 95% CI, 0.403–1.810; *P* = 0.67; **Figure 4F**). In summary, the IMiD-based therapy affected the prognosis of myeloma in patients with high CRBN expression; however, it did not affect the prognosis of patients with low CRBN expression.

### Predictors of CRBN Expression

**Table 2** shows the results of the logistic regression analyses for predicted high CRBN expression. The univariable analyses revealed that high CRBN expression was significantly associated with female sex, higher percentage of bone marrow plasma cells, higher serum M-protein level, high serum FLC ratio (≤0.01 or ≥100), and higher β2-microglobulin level. Multivariable analysis using the backward stepwise elimination method and including all the variables assessed in the Univariable analysis (age, sex, ECOG performance status, percentage of bone marrow plasma cells, serum M-protein, serum FLC ratio, β2-microglobulin, LDH, cytogenetic risk factor, and R-ISS stage) confirmed that high CRBN expression was independently predicted by female sex (Odds ratio [OR], 2.969; 95% CI, 1.351–6.522; *P* = 0.007), higher serum M-protein level (OR, 1.311; 95% CI, 1.108–1.551; *P* = 0.002), high serum FLC ratio (OR, 3.590; 95% CI, 1.647–7.823; *P* = 0.001), and higher β2-microglobulin level (OR, 1.104; 95% CI, 1.007–1.210; *P* = 0.034). Based on these results, we constructed a new nomogram model to predict CRBN expression in myeloma patients.

**Table 2:** Univariable and Multivariable analyses for high CRBN expression prediction.

Prognostic factors	Univariable			Multivariable		
	OR	95% CI	*P*	OR	95% CI	*P*
Age, years	0.977	0.944, 1.012	0.199			
Sex, female	2.410	1.229, 4.727	0.010	2.969	1.351, 6.522	0.007
ECOG performance status, ≥2	1.327	0.341, 5.158	0.683			
BM plasma cells, %	1.022	1.008, 1.035	0.001			
Serum M-protein, mg/dL	1.232	1.065, 1.426	0.005	1.311	1.108, 1.551	0.002
Serum FLC ratio, ≤0.01 or ≥100	3.277	1.644, 6.534	0.001	3.590	1.647, 7.823	0.001
ß2-microglobulin, mg/L	1.094	1.007, 1.173	0.031	1.104	1.007, 1.210	0.034
LDH, ≥ upper normal range	0.847	0.432, 1.660	0.628			
Cytogenetics, high-risk*	1.772	0.829, 3.788	0.140			
R-ISS, Stage III	1.598	0.715, 3.571	0.254			

BM, bone marrow; ECOG, Eastern Cooperative Oncology Group; FLC, free light chain; LDH, lactate dehydrogenase; OR, Odds ratio; R-ISS, Revised International Staging System.

*High-risk cytogenetics was defined as t(4;14), t(14;16), del(17/17p), TP53 deletion, or chromosome 1 abnormalities including gain(1q) and del(1p).

### Predictive Nomogram Model

**Figure 5:**
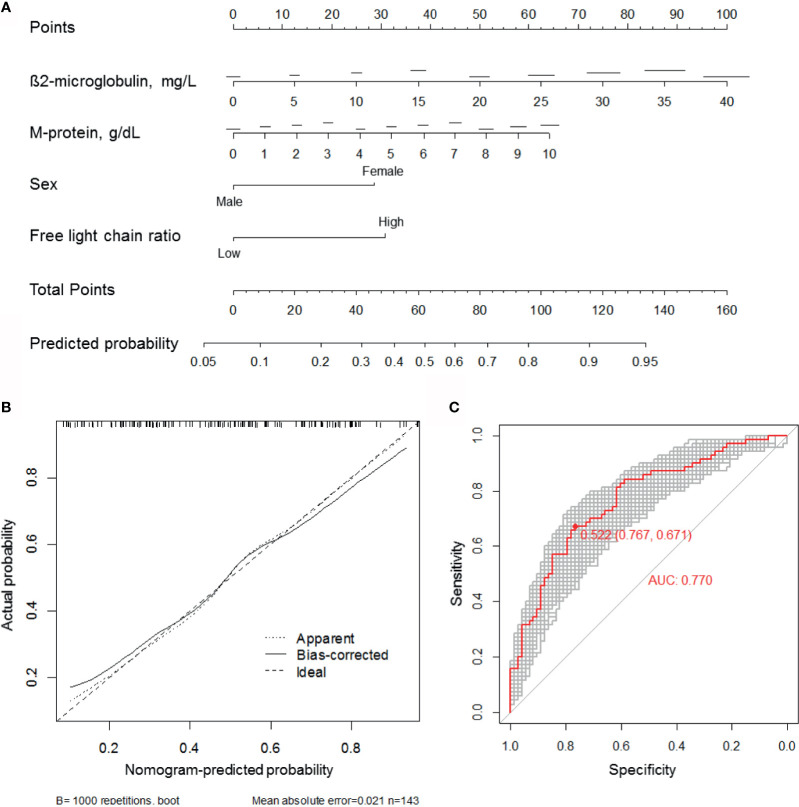
Development of the CRBN prediction model. **(A)** A nomogram for estimating high (H-score ≥6) CRBN expression. **(B)** Calibration curves were created by plotting the actual probabilities (y-axis) against the nomogram-predicted probabilities (x-axis). Apparent model was constructed based on the original data. The bias-corrected model was obtained by subtracting the optimism estimate from the apparent performance. The optimism estimate was calculated as the difference between bootstrap performance with 1,000 repetitions and the test performance. Test performance was obtained by applying the model to the original data without any modification. The 45-degree line indicates an ideal model. **(C)** ROC curves and AUC analyses with 1,000 bootstrap replicates for evaluating the performance and prediction accuracy of the nomogram model. AUC, area under the curve; ROC, receiver operating characteristic.

We constructed a predictive nomogram model based on the scores for β2-microglobulin, serum M-protein, sex, and serum FLC ratio, with higher scores predicting a higher CRBN expression (**Figure 5A**). The calibration plots for high CRBN expression probabilities showed good agreement between the actual and predicted outcomes (**Figure 5B**). ROC curve and AUC analyses were performed with 1,000 bootstrap replications to evaluate the performance of the nomogram (**Figure 4C**). We assessed the performance as being good based on the cross validated AUC value of 0.770. The optimal cut-off point for the estimated high CRBN expression was 0.522 (64.2 points), with a sensitivity of 0.767 and specificity of 0.671. Ultimately, we estimated and validated prognostic value of the new nomogram model by using the internal and external datasets.

### Prognostic Implications of the CRBN Prediction Model

**Figure 6:**
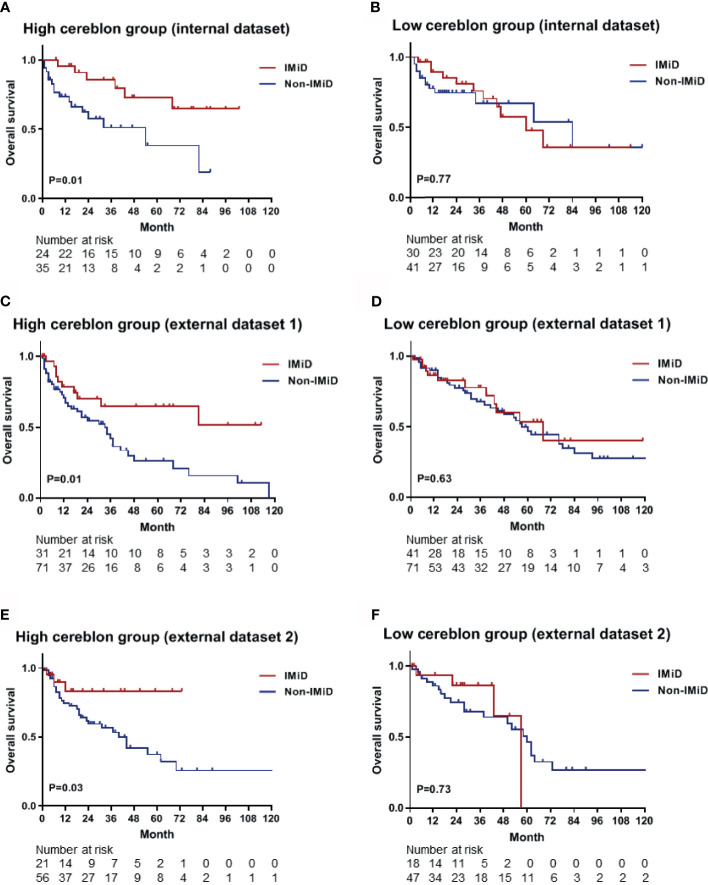
Kaplan–Meier survival curves for OS in the high and low CRBN groups based on the CRBN prediction model. Internal dataset (data used in model development) was used for analysis and two external datasets (data from two other hospitals) were used for validation. **(A)** The OS curves for the high CRBN (n = 59) and **(B)** low CRBN (n = 71) groups from the internal dataset. The OS curves for **(C)** the high (n = 102) and **(D)** low (n = 112) CRBN groups from the external dataset 1 and for **(E)** the high (n = 77) and **(F)** low (n = 65) CRBN groups from the external dataset 2. Patients exhibiting high levels of CRBN and receiving IMiD treatment showed significantly longer OS than those receiving non-IMiD treatment. No significant differences were found between IMiD and non-IMiD treatment in the low CRBN groups. IMiD, immunomodulatory drug; OS, overall survival.

Based on calculated CRBN expression levels, the Kaplan-Meier survival analysis for OS was performed using the predictive nomogram model (**Figure 5**). The high CRBN group was defined as >64.2 points in the nomogram model. The survival outcomes in the internal dataset were evaluated and two external datasets were used for validation. First, the patients from the internal dataset were examined. In the high CRBN group, patients who received IMiD-based therapy had a significantly longer OS (*P* = 0.01; **Figure 6A**), compared to patients who received non-IMiD-based therapy. However, in the low CRBN group, no significant difference in the OS was observed following the IMiD therapy (*P* = 0.77; **Figure 6B**). Second, the patients from the external dataset 1 were examined. In the high CRBN group, patients who received IMiD-based therapy had significantly longer OS (*P* = 0.01; **Figure 6C**), compared to that in patients who received non-IMiD-based therapy. However, in the low CRBN group, no significant differences in the OS were observed following IMiD therapy (*P* = 0.63; **Figure 6D**). Finally, patients from the external dataset 2 were examined. In the high CRBN group, patients who received IMiD-based therapy had a significantly longer OS (*P* = 0.03; **Figure 6E**). However, in the low CRBN group, no significant differences in the OS were observed following IMiD therapy (*P* = 0.73; **Figure 6F**). The PFS analyses results are presented in the **Supplementary Figure 1**. The high CRBN group of patients who received IMiD-based therapy showed longer PFS in the internal dataset (*P* = 0.03; **Supplementary Figure 1A**), external dataset 1 (*P* = 0.05; **Supplementary Figure 1C**), and external dataset 2 (*P* = 0.08; **Supplementary Figure 1E**). However, no significant difference in the PFS was observed between the IMiD-based and non-IMiD-based therapy in the internal dataset (*P* = 0.98; **Supplementary Figure 1B**), external dataset 1 (*P* = 0.10; **Supplementary Figure 1D**), and external dataset 2 (*P* = 0.26; **Supplementary Figure 1F**). In summary, in the high CRBN group, IMiD-based therapy was associated with significantly longer OS and PFS, while in the low CRBN group, no significant differences were observed between the IMiD-based and non-IMiD-based therapy groups.

## Discussion

This study aims to develop a prediction model for CRBN expression using blood markers from multiple myeloma patients. Our analysis showed that high CRBN expression in bone marrow plasma cells was independently predicted by female sex, high serum FLC ratio, and higher serum M-protein, and β2-microglobulin levels. Based on these results, we constructed a new nomogram model to predict high CRBN expression in patients with multiple myeloma. Our model was able to accurately detect high CRBN expression and identify patients who mostly benefited from IMiD-based therapy.

Several studies evaluated the prognostic value of CRBN in patients with multiple myeloma (10–16). CRBN is expressed in bone marrow plasma cells of multiple myeloma patients (16), and patients with higher CRBN expression showed favorable prognosis following IMiD-based therapy (12–16, 19). Thus, CRBN expression might have IMiD efficiency predictive value in multiple myeloma patients, although there is controversy regarding the appropriate method for measuring CRBN expression (20, 21). Some groups used tissue microarrays and fluorescence IHC to quantify CRBN expression (22), and other groups correlated the CRBN expression and the outcomes following IMiD therapy using gene expression profiling (13–15). Although the evaluation of CRBN expression to predict individual patient response to IMiD therapy may represent an important step in determining the treatment strategy, this evaluation should be standardized. For instance, due to the complexity of CRBN gene expression regulation, performing a single RT-PCR is insufficient for accurate measurements (20). Several CRBN isoforms produced by alternative mRNA splicing are reported in multiple myeloma (20, 23) and standardized RT-PCR methods have not been proposed yet. The IHC method may offer advantages over molecular methods that do not require cell purification, inexpensive nature, and lab availability; thus, it can provide cost-effective predictive value in multiple myeloma (16). Moreover, the cell morphology is conserved while using IHC, allowing it to recognize immunostaining heterogeneity and determine in which subcellular compartment the confirmed positivity is localized. Thus, we used IHC to detect and evaluate CRBN expression. However, as previously described, there are no approved tests and methods for CRBN expression measurement and a previous study reported a lack of correlation between CRBN protein and mRNA levels (23).

The benefit of IMiD therapy related to CRBN expression in multiple myeloma patients has also been addressed in several previous studies. One study reported that CRBN and IKZF1 gene expression is significantly associated with longer OS in patients treated with pomalidomide (10). A previous study showed that longer PFS is observed in patients exhibiting high levels of CRBN and treated with thalidomide (median, 22 months *vs*. not reached) than those exhibiting low levels of CRBN, while no association was found between PFS and CRBN levels when the patients were treated with bortezomib (12). Furthermore, a study using thalidomide maintenance therapy suggested that CRBN expression is significantly associated with longer PFS, but not OS (14). A study reporting the effect of lenalidomide treatment on multiple myeloma patients indicated that PFS is significantly shorter in patients exhibiting low CRBN levels than in those exhibiting high CRBN levels (5.6 *vs*. 19.7 months) and a similar effect was observed for OS (11.4 *vs*. 30.5 months) (15). However, one study reported that CRBN expression does not correlate with PFS or OS in patients treated with lenalidomide (13). A previous study which measured the CRBN expression using IHC indicated that the CRBN expression is an independent factor associated with the treatment response of lenalidomide and thalidomide; however, CRBN does not affect survival outcomes including PFS (median, 8 *vs*. 8 months) and OS (median, NR *vs*. 27 months) in both lenalidomide and thalidomide groups. In the present study, the high CRBN patients treated with IMiDs had a PFS median of 29 months compared to the 10 months PFS median observed in those who received non-IMiD therapy. The median OS was NR and 54 months in the IMiD and non-IMiD groups, respectively. As a result, IMiD therapy was strongly associated with favorable PFS and OS in the high CRBN group, but not the low CRBN group. We believe that the differences between the previous results and our outcomes might be explained by the lack of approved antibodies for CRBN protein and reliable detection methods for CRBN expression. We analyzed newly diagnosed multiple myeloma patients, measured their CRBN levels using IHC and a commercial antibody, and showed that the IMiD therapy affects patient survival in a CRBN expression-dependent manner. Therefore, prediction strategies for high CRBN expression might help identify patients who might benefit from IMiD therapy and could improve the clinical outcomes of multiple myeloma patients.

Nomogram models are generally more accurate than risk group-based models and can contain uncategorized continuous variables. Statistical formulas can contain more information than nomogram; however, these are inconvenient to use in a clinical setting (24). We developed a predictive nomogram model which showed good calibration, as indicated by the actual and nomogram-predicted probabilities. A bootstrap aggregating method was used to avoid overfitting and enhance the accuracy of calibration. AUC analyses were also performed with 1,000 bootstrap replications to evaluate the performance of the nomogram. We observed a good performance based on the AUC value (0.770) and the sensitivity (0.767) as well as the specificity (0.671) of the model. Next, we applied this model to internal and external datasets and observed that the predictions produced by the nomogram model were comparable to the measured CRBN expression. For the internal dataset, both the nomogram-predicted high CRBN group and the measured high CRBN group were characterized by a median PFS of 29 months in the IMiD group and 10 months in the non-IMiD group, while the median OS was NR in the IMiD group and 54 months in the non-IMiD group. For the validation analyses using the two external datasets, the high CRBN group had a median PFS of 16 (external dataset 1) and 23 months (external dataset 2) in the IMiD group and 8 (external dataset 1) and 10 (external dataset 2) months in the non-IMiD group. Furthermore, the median OS was NR (external dataset 1 and 2) in the IMiD group and 34 (external dataset 1) and 40 (external dataset 2) months in the non-IMiD group. The survival outcomes obtained from the internal dataset were comparable to those from the external datasets and showed that IMiD-based therapy was associated with significantly longer OS and PFS in the validation analyses. Therefore, our nomogram prediction model can be used as a prognostic tool for personalized treatment approaches and applications including IMiD therapy in multiple myeloma.

There are several limitations to this study. First, the current study was conducted using a retrospective analysis and included a relatively small sample size. Thus, any confirmatory conclusions cannot be drawn from the results of this study. Second, no study has reported a cut-off value for high or low CRBN expression measured using IHC. Moreover, the most appropriate testing method for measuring CRBN expression is not confirmed in the multiple myeloma patients. Although we have used study-defined value as the cut-off points for the IHC values, a more reliable method is required to determine an appropriate cut-off point and establish a standardized CRBN detecting method. Further studies could be required for optimizing the detection protocols in multiple myeloma.

In conclusion, the present study showed that IMiD therapy might have a clinical benefit in multiple myeloma patients exhibiting high CRBN expression. Furthermore, we developed a predictive nomogram model for high CRBN expression based on clinically significant blood markers. This nomogram could improve the prognostic evaluation of the efficiency of IMiD therapy in myeloma patients with high CRBN expression and might provide personalized initial treatment strategies to clinicians.

## Data Availability Statement

The original contributions presented in the study are included in the article/**Supplementary Material**. Further inquiries can be directed to the corresponding author.

## Ethics Statement

The studies involving human participants were reviewed and approved by the institutional review board of the Korea University Medical Center. The patients/participants provided their written informed consent to participate in this study.

## Authors Contributions

BHL and BSK proposed the study concept and design. KWK, MJJ, and ESY contributed to the patient data collection and sample preparation. BHL performed the experiments and analyzed the data. DSK, SRL, HJS, YP, and CWC helped with data interpretation. BHL wrote the draft of the manuscript. BSK revised the manuscript. All authors contributed to the article and approved the submitted version.

## Funding

This research was supported by the Bio and Medical Technology Development Program of the National Research Foundation of Korea (NRF), funded by the Ministry of Science and ICT (NRF-2017M3A9C8060403).

## Conflict of Interest

The authors declare that the research was conducted in the absence of any commercial or financial relationships that could be construed as a potential conflict of interest.
