# Supplementary material for: Prediction Model for Cereblon Expression in Bone Marrow Plasma Cells Based on Blood Markers in Multiple Myeloma Patients
Source: Front Oncol. 2021 Jul 14;11:687361. doi: 10.3389/fonc.2021.687361 (PMC8316857; doi:10.3389/fonc.2021.687361)
Supplement: Supplementary file 1 [file DataSheet_1.docx]

Supplementary Material

# Supplementary Table

**Supplementary Table 1.** Expression level of CRBN protein by ELISA

| **Sample** | **Value** | **Results** | **Mean Results** | **SD** | **CV** |
| --- | --- | --- | --- | --- | --- |
| #1 | 0.044  0.045 | 0.196  0.204 | 0.200 | 0.005 | 2.7 |
| #2 | 0.065  0.051 | 0.333  0.245 | 0.289 | 0.062 | 21.4 |
| #3 | 0.038  0.034 | 0.159  0.137 | 0.148 | 0.015 | 10.3 |
| #4 | 0.137  0.077 | 0.786  0.405 | 0.595 | 0.270 | 45.3 |
| #5 | 0.084  0.060 | 0.451  0.299 | 0.375 | 0.108 | 28.8 |
| #6 | 0.045  0.032 | 0.207  0.125 | 0.166 | 0.058 | 35.0 |
| #7 | 0.022  0.018 | 0.061  0.034 | 0.048 | 0.019 | 39.5 |
| #8 | 0.054  0.046 | 0.264  0.210 | 0.237 | 0.038 | 16.1 |
| #9 | 0.022  0.015 | 0.060  0.016 | 0.038 | 0.031 | 81.8 |
| #10 | 0.106  0.098 | 0.590  0.540 | 0.565 | 0.035 | 6.3 |
| #11 | 0.062  0.052 | 0.311  0.246 | 0.278 | 0.046 | 16.5 |
| #12 | 0.093  0.082 | 0.508  0.437 | 0.472 | 0.050 | 10.7 |

# Supplementary Figure


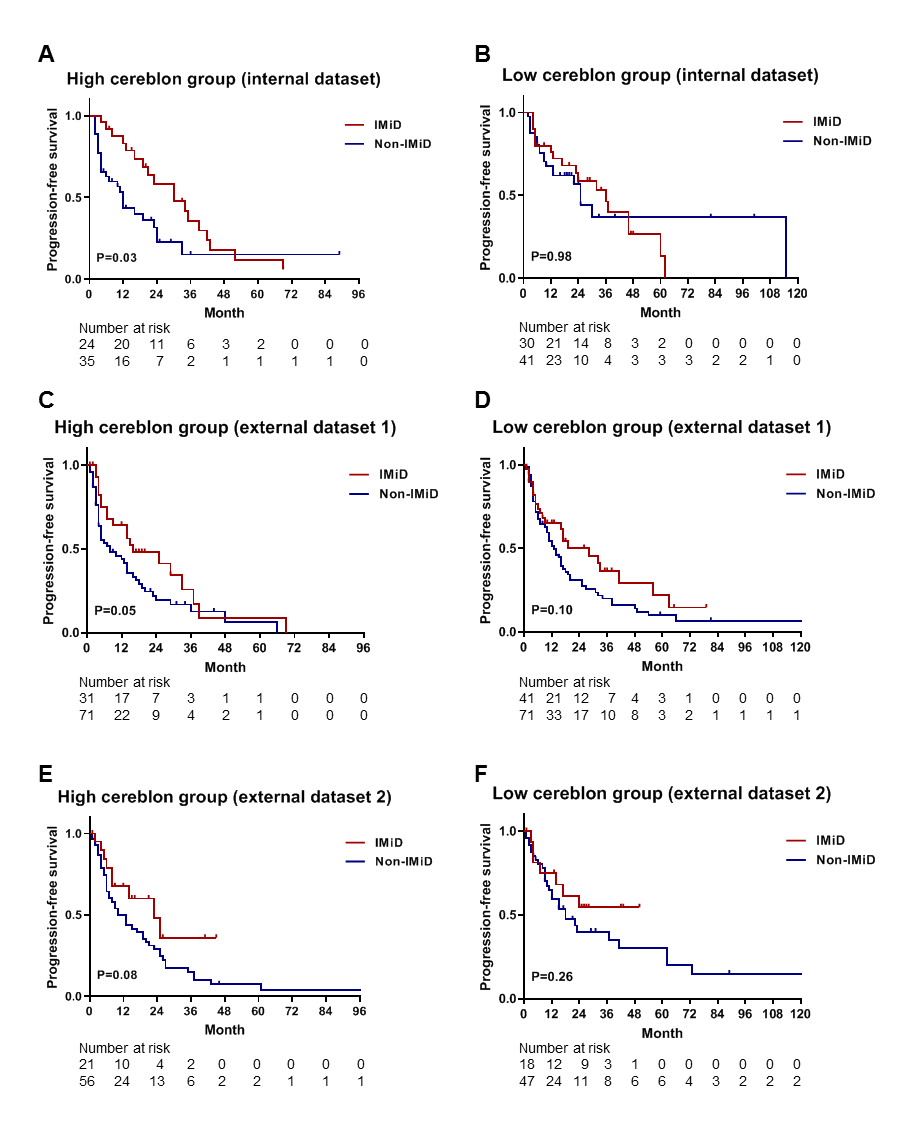


**Supplementary Figure 1.** Kaplan–Meier survival curves for PFS in the high and low CRBN groups based on the CRBN prediction model. Internal dataset (data used in model develop) was used for analysis and two external datasets (data from two other hospitals) were used for validation. (A) The PFS curves in the high CRBN (n = 59) and (B) low CRBN (n = 71) groups from the internal dataset. The PFS curves for (C) the high (n = 102) and (D) low (n = 112) CRBN groups from the external dataset 1 and for (E) the high (n = 77) and (F) low (n = 65) CRBN groups from the external dataset 2. Patients exhibiting high levels of CRBN and receiving IMiD treatment showed significantly longer PFS than those receiving non-IMiD treatment. No significant differences were found between IMiD and non-IMiD treatment in the low CRBN groups. IMiD, immunomodulatory drug; PFS, progression-free survival.
